# Prevalence of Interstitial Lung Abnormalities (ILAs) in Italian Lung Cancer Screening Programs: A Monocentric Study

**DOI:** 10.3390/jcm15093193

**Published:** 2026-04-22

**Authors:** Diletta Cozzi, Caterina Giannessi, Luca Gozzi, Edoardo Cavigli, Chiara Moroni, Giulia Picozzi, Katia Ferrari, Vittorio Miele

**Affiliations:** 1 Department of Emergency Radiology, Careggi University Hospital, 50134 Florence, Italy; giannessic@aou-careggi.toscana.it (C.G.); lucagozzi1@gmail.com (L.G.); edoardocavigli@yahoo.it (E.C.); chiaramoroni73@gmail.com (C.M.); vittorio.miele@unifi.it (V.M.); 2 Institute for Cancer Research, Prevention and Clinical Network (ISPRO), 50139 Florence, Italy; g.picozzi@ispro.toscana.it; 3 Respiratory Medicine Unit, Careggi University Hospital, 50134 Florence, Italy; ferrarik@aou-careggi.toscana.it; 4 Department of Experimental and Clinical Biomedical Sciences “Mario Serio”, University of Florence, 50134 Florence, Italy

**Keywords:** lung cancer, screening, low-dose CT, interstitial lung abnormalities, interstitial lung disease

## Abstract

**Background**: Because of the increased awareness of the clinical importance of ILAs on chest CT, this study aims to determine the prevalence of ILAs in an Italian health cohort undergoing CT for a lung cancer screening (LCS) program and quantify ILA using both visual and deep learning-based analyses. **Methods**: In this observational, retrospective monocentric study, 500 participants (ITALUNG2, *n* = 100; RISP, *n* = 400) underwent low-dose CT (CTDI < 2mGy). Two radiologists retrospectively reviewed the images and determined the presence and extent of ILAs, classifying them as fibrotic or non-fibrotic, while a lung texture analysis was performed by using commercially available deep learning–based software. **Results**: ILAs were present in 34 patients (11 females and 23 males), with a prevalence of 6,8%, with similar rates across both screening cohorts taken individually. Interobserver agreement between radiologists was almost perfect, whereas concordance between visual and automated quantification was substantial. Visual assessment tended to yield higher estimates of ILA extent compared with software-based analysis. **Conclusions**: These findings confirm that ILAs are relatively common in LCS populations and highlight the importance of their detection and characterization, particularly for fibrotic patterns. Differences between visual and automated approaches underline the need for further refinement and validation of quantitative tools.

## 1. Introduction

With the rapid advancement of computed tomography (CT) technology and the widespread implementation of lung cancer screening (LCS) programs, incidental findings beyond lung cancer are receiving increasing attention. Among these, interstitial lung abnormalities (ILAs) have emerged as a clinically relevant entity due to their potential association with early interstitial lung disease (ILD) and adverse outcomes.

ILAs are radiological findings identified on CT scans in individuals without a prior diagnosis of ILD who underwent full-chest CT or partial chest imaging (e.g., abdominal or cardiac CT including the lower-lung zones) for other reasons, in which abnormalities have been found involving more than 5% of any lung zone (upper, middle, or lower—delimited by the inferior aortic arch and the right inferior pulmonary vein) [1,2]. According to pattern distribution, ILAs are defined as subpleural or central-predominant in the axial plane and upper- or lower-lung-predominant in the craniocaudal plane [1,2]. These abnormalities include ground-glass or reticular patterns, lung distortion, traction bronchiectasis or bronchiolectasis, honeycombing, and non-emphysematous cysts, as described in the Fleischner Society Glossary of Thoracic Imaging. Although a ≥5% extent threshold has been proposed for classification, this cutoff remains partly arbitrary and largely based on visual assessment, highlighting the need for more objective and reproducible quantification methods [3].

Recent studies have demonstrated substantial variability in the prevalence of ILAs across different populations. It is worth noting that although most of the literature agrees on a pooled prevalence of approximately 7% in the general population, as reported in the recent meta-analysis by Grant-Orser et al., these data largely derive from selected research cohorts that may be biased by inclusion criteria conferring a higher risk of ILAs [4]. This limitation was highlighted in a recent study by Sverzellati et al., in which more than 20,000 patients undergoing either abdominal or thoracoabdominal CT for various clinical indications were evaluated for ILAs, with a prevalence of only 1.7% [5]. Notably, 43.9% of ILAs had not been originally reported, the majority being subpleural non-fibrotic lesions (55%). This high percentage is clinically relevant, as interstitial abnormalities have been shown to be associated with accelerated decline in lung function, increased mortality, and a higher risk of developing lung cancer with unfavorable prognosis [5,6,7,8,9].

In smokers and lung cancer screening cohorts, ILA prevalence has been reported in the range of 4–17%, with a pooled prevalence of around 7% [4,8,10,11,12,13,14]. Lower prevalence has been observed in Asian populations compared to Western populations, as documented by Tsushima et al. and Chae et al. [15,16]. This wide variability may be attributable to several factors, including age, genetic predisposition (such as the MUC5B promoter polymorphism), environmental exposures, and differences in imaging protocols [14,17,18].

Despite most patients with ILAs being asymptomatic, it is mandatory to recognize this finding when present, specifically the subpleural fibrotic form, since it has been associated with accelerated lung function decline, a higher likelihood of progression toward clinically significant ILD and thus increased all-cause mortality, and a greater risk of lung cancer development and progression [19].

In recent years, artificial intelligence (AI) and deep learning techniques have been increasingly applied to thoracic imaging. Recent studies have explored automated approaches for lung texture analysis, aiming to provide quantitative and reproducible assessments of ILAs. While these tools show promising diagnostic performance, their clinical integration is still challenged by variability in acquisition protocols, differences in population characteristics, and the lack of universally accepted thresholds.

In this context, our monocentric study—conducted within the Italian Lung Cancer Screening (LCS) Program at our University Hospital—aims to assess the prevalence of ILAs in our screening cohort, compare visual assessment with deep learning-based quantification and evaluate interobserver agreement between radiologists. By integrating traditional radiological evaluation with AI-based analysis, this study seeks to contribute to the ongoing effort to standardize ILA detection and improve its clinical interpretation.

## 2. Materials and Methods

### 2.1. Patient Population and Study Design

In this retrospective study, we included 500 consecutive patients recruited in two LCS trials. The first cohort comprised 100 individuals enrolled in the ITALUNG2 LCS trial, identified and declared eligible by general practitioners, living in the Florence district, aged 55–75 years, and with a smoking history of ≥30 pack-years within the previous 10 years.

The second cohort consisted of 400 volunteers living in the Florence district, recruited through advertisements, a toll-free number, or a dedicated website, and enrolled in the Italian Pulmonary Screening Network (RISP), part of the European 4-IN-THE-LUNG-RUN project. Inclusion criteria were age 55–75 years, a smoking history of ≥30 pack-years, and either current smoking or smoking cessation within the last 15 years.

The exclusion criteria for both screening trials were the same: recent abnormal pulmonary findings under clinical evaluation; chest CT within the previous year; current or prior lung cancer; prior malignancy other than non-melanoma skin cancer; and inability to provide informed consent (Table 1). All participants provided written informed consent and completed a structured questionnaire on medical history (cardiovascular disease (CVD), chronic bronchitis, emphysema), smoking history (current or former status, years since cessation, pack-years), and comorbidities (COPD, CVD). Those who met the criteria proceeded to LDCT.

### 2.2. LDCT Acquisition and Imaging Analysis

We analyzed 500 baseline low-dose non-contrast-enhanced CT (LDCT) examinations obtained between October 2022 and May 2023. All CT examinations were performed using the same spiral CT scanner (Somatom Sensation 128; Siemens Medical Solutions, Forchheim, Germany) and the same protocol (scan of the whole lung in full inspiration) with standardized scanning parameters (1 mm slice thickness, 120 kV voltage, 40 mAs tube current, collimation ≤ 1 mm) (Table 2). The radiation dose of a screening chest LDCT varies depending on the subject’s biometric features (height and weight), but CTDI was <2.0 mGy for all participants. Before the assessment, the quality of LDCT examination images was considered adequate or inadequate due to artifacts caused by motion, the presence of metallic stents, etc.

**Figure 1:**
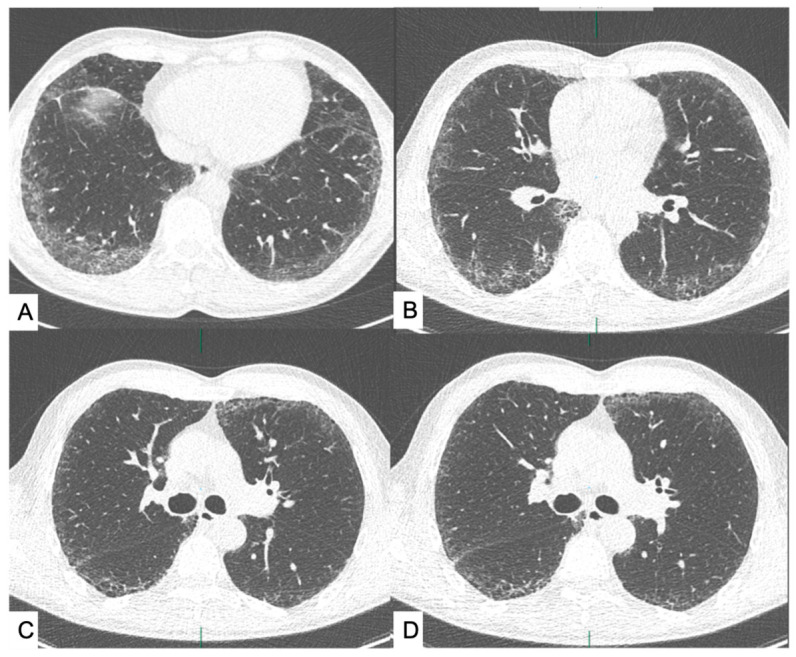
Examples of ILAs defined as non-dependent ground-glass abnormality affecting ≥5% of any lung zone (**A**) or non-dependent reticular abnormality, honeycombing, traction bronchiectasis, nonemphysematous cysts, and architectural distortion (**B**–**D**); low-dose CT axial views.

Two thoracic radiologists, with 10 and 5 years of experience respectively, independently reviewed all LDCT scans in random order and without access to clinical information. Lung nodules were reported according to Lung-RADS (v2022) criteria, and the presence of coronary calcifications and emphysema was also recorded as absent (0) or present (1). ILAs were assessed following the Fleischner Society guidelines and were defined as non-dependent ground-glass or reticular abnormalities involving ≥5% of any lung zone as well as honeycombing, traction bronchiectasis, non-emphysematous cysts, or architectural distortion (Figure 1) [3]. ILAs were then classified into two subtypes, fibrotic and non-fibrotic, following the latest American Thoracic Society (ATS) Statement [2]. For each case, both radiologists provided a visual estimate of ILA extent. In instances of disagreement, consensus was reached through joint review. This consensus evaluation served as the reference standard for assessing the diagnostic performance of the quantitative software.

### 2.3. Deep Learning Analysis

**Figure 2:**
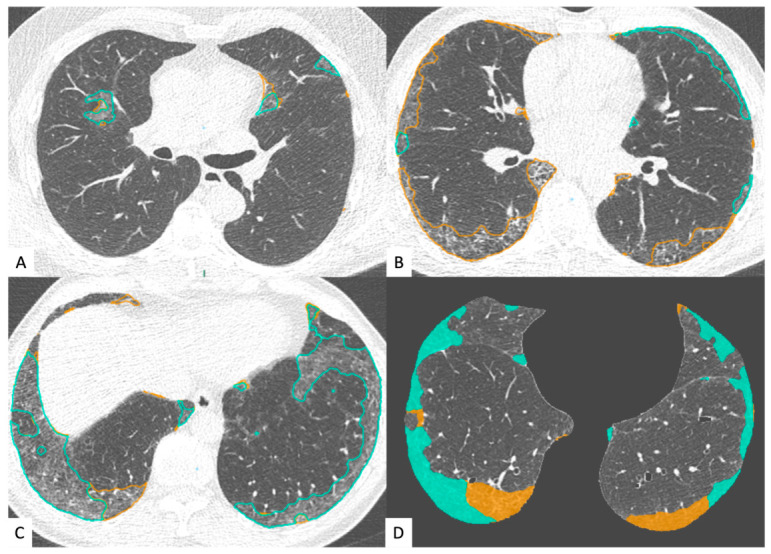
AVIEW Lung Texture interface showing ILA’s subtypes and segmentation; blue—non-fibrotic ILA; yellow—fibrotic ILA. Figures in (**A**,**C**) show mostly non-fibrotic ILA, while figure (**B**) shows mostly fibrotic ILA. Image (**D**) is an example of the software user interface.

**Figure 3:**
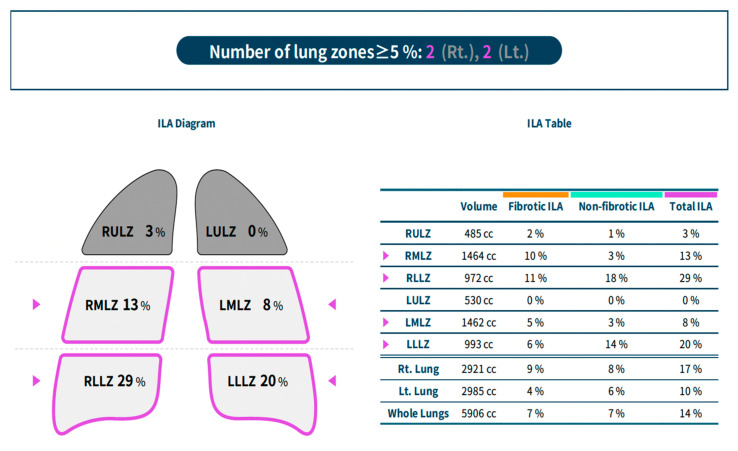
AVIEW Lung Texture ILA, version 1.1.39.14; Coreline Soft user interface, showing global extension of ILA and extension in every lung lobe.

We analyzed lung texture by using commercially available deep learning-based lung texture analysis software for ILA (AVIEW Lung Texture ILA, version 1.1.39.14; Coreline Soft), which was developed by modifying a deep learning-based algorithm for lung segmentation and pattern classification for ILA [20,21]. This software uses a two-dimensional U-Net architecture to segment abnormal lung lesions and provides a quantitative percentage analysis (total ILA and ILA of individual lobes). It classifies ILA as follows: (a) fibrotic; (b) non-fibrotic. The software interface with ILA segmentation is shown in Figure 2 (blue—non-fibrotic ILA; yellow—fibrotic ILA). The overall ILA burden was calculated as the sum of both non-fibrotic and fibrotic components. The software classified a chest CT as positive for ILA when the total extent reached ≥5% in at least one of the six lung zones, with results displayed directly on the user interface. Figure 3 illustrates the software interface, highlighting both the overall ILA extent and its distribution across individual lobes. The performance of this system has been validated using LDCT in a recent published study [22].

### 2.4. Statistical Analysis

Unless otherwise specified, all data were expressed as mean ± standard deviation (SD). Data were summarized as numerical and categorical variables. Descriptive statistical analysis was applied in our study. Interobserver agreements were assessed with using Cohen’s κ coefficient and classified as follows: “less than a chance” (κ < 0); “slight” (κ = 0.01–0.20), “fair” (κ = 0.21–0.40), “moderate” (κ = 0.41–0.60), “substantial agreement” (κ = 0.61–0.80), or “almost perfect agreement” (κ = 0.81–1.00).

## 3. Results

Within our cohort of 500 patients, ILAs were identified in 34 individuals corresponding to an overall prevalence of 6.8%. Among these, 23 were male (67%, 20 RISP and 3 ITALUNG2) and 11 were female (32%, 8 RISP and 3 ITALUNG2), with a mean age of 63.9 years (range, 55–74 years). Furthermore, these subjects had an average of 25 pack-years and the following comorbidities: 26 (76.5%) with coronary artery calcifications (CACs) and 18 (52%) with COPD and/or emphysema.

When stratified by screening program, the prevalence of ILAs was comparable between cohorts, being 7% (28/400) in the RISP group and 6% (6/100) in the ITALUNG2 group, indicating a consistent distribution of findings despite differences in recruitment strategies (Table 3). Regarding radiological ILA patterns, 13 patients (38%) presented non-fibrotic ILAs, while the majority (21 patients, 62%) had the fibrotic subtype. This predominance of fibrotic features is clinically relevant given their known association with disease progression. Notably, 52% of patients with ILAs also had emphysema and 76.5% of these also had CACs, suggesting a frequent overlap between interstitial involvement, obstructive smoking-related lung damage and cardiovascular involvement.

In addition, 17.3% of patients with ILAs had a positive screening test (defined as Lung—RADS v2022: 4A—4B—4X). The extent of ILAs for each patient was assessed by two thoracic radiologists via visual evaluation and using an automated AI-based software. Interobserver agreement between the two radiologists for ILA detection and classification was almost perfect (κ = 0.83), supporting the reliability of visual assessment in this setting. In contrast, agreement between visual estimation and automated software quantification was substantial but lower (κ = 0.62). Notably, visual evaluation consistently yielded higher estimates of ILA extent compared with the deep learning-based analysis (Table S1), suggesting a systematic difference between subjective and automated approaches.

## 4. Discussion and Conclusions

The present study demonstrates that ILAs are a relatively frequent finding in individuals undergoing lung cancer screening, with a prevalence of 6.8% in our cohort. This value aligns with estimates reported in the recent literature for high-risk populations, particularly among older smokers, supporting the external validity of our findings. The comparable prevalence observed between the two screening programs further strengthens the robustness of the results despite differences in recruitment pathways [4,14,23].

Our findings highlight the importance of detecting and classifying ILAs in the setting of LCS, particularly the fibrotic subtype, which represented nearly two-thirds of all detected abnormalities; this is the one more likely to progress and is proven to be associated with increased mortality [2,12,24]. It is already known that ILAs are associated with worsening of respiratory symptoms, loss of lung function, radiologic progression of lung fibrosis and increased all-cause mortality, as recently further stated by ATS where it is remarked that a systematic assessment and documentation of the presence of ILAs in smokers is needed so that selected patients may be referred to a dedicated pneumological evaluation [2].

Moreover, more than half of the patients with ILAs in our cohort also had emphysema. This coexistence is clinically relevant and reflects the complex spectrum of smoking-related lung injury. Previous studies have suggested that this overlap may influence functional impairment, sometimes masking the expected decline in pulmonary function typically associated with emphysema alone. This interaction highlights the need for integrated radiological and clinical evaluation in this population [25].

From a methodological perspective, our results confirm excellent interobserver agreement between radiologists, indicating that visual assessment remains a reliable approach when performed by experienced readers. However, the only moderate-to-substantial agreement between visual and automated quantification points to current limitations of deep learning tools. In particular, the systematic overestimation observed with visual analysis suggests that subjective evaluation may be influenced by pattern recognition biases, while automated methods may be more conservative due to predefined thresholds [16,22,26,27]. In relation to that, several studies have proposed alternative thresholds to improve the performance of automated detection. For instance, Choi et al. [27] reported a median cutoff of 3.8% in the Multi-Ethnic Study of Atherosclerosis, Kim et al. suggested 3.6% [22], and Mathai et al. [26] identified 1.8% as optimal for detecting preclinical fibrosis in first-degree relatives of patients with familial interstitial pneumonia. Similarly, in the Korean lung cancer screening program, the same software for deep learning-based texture analysis we used in our study (ndr. AVIEW Lung Texture ILA, version 1.1.39.14; Coreline Soft) achieved high sensitivity and specificity for ILA detection using a threshold of 1.8% lung area involvement [16]. This evidence illustrates how, despite the promising role of artificial intelligence in assisting radiologists and providing quantitative insights, several limitations persist. Automated quantification remains highly sensitive to imaging variability, including technical factors such as inspired lung volumes, CT acquisition parameters (particularly in LDCT for LCS), reconstruction algorithms, and scanner models [28,29]. The performance of deep learning models is highly dependent on the quality and representativeness of training datasets, and issues such as overfitting and limited generalizability continue to be relevant [30,31,32]. In addition, the morphological features of ILAs—often subtle, heterogeneous, and subpleural—pose further challenges for automated detection, particularly with regard to accurate segmentation from the chest wall.

This study has some limitations. First, its retrospective design may have limited the ability to ascertain whether ILAs were truly absent before baseline CT. Second, and most importantly, the study population was unbalanced, as 400 patients were enrolled from the RISP trial and only 100 from ITALUNG2. Although this disparity did not significantly affect the present analysis, it could become statistically relevant in larger cohorts given the different recruitment strategies used in the two screening programs [33]. Further research is warranted with more homogeneous sample sizes across screening trials. Finally, nearly all participants were Caucasian and resided in the Florence district, limiting the generalizability of our findings. In light of these limitations, additional multicohort studies are needed to assess the robustness and external validity of automated approaches. Advances in artificial intelligence and deep learning hold promise for overcoming current challenges, but rigorous validation and threshold optimization will be essential before these tools can be widely implemented in clinical practice.

To conclude, despite our results demonstrate excellent interobserver agreement between radiologists and a prevalence consistent to rates reported in the literature in smokers and in LCS trials, discrepancies between visual and automated quantification highlight the current limitations of AI tools, which tend to underestimate disease extent. This finding is in line with recent studies showing that AI-based analyses often yield results that differ from radiologists’ visual assessments. In the context of rapidly evolving technologies and their increasing integration into clinical practice, these discrepancies suggest the need to reconsider current diagnostic criteria, potentially adjusting detection thresholds to enhance the accuracy and reliability of automated ILA identification.

## Figures and Tables

**Table 1:** Inclusion and exclusion criteria to participate in LCS trials.

Category	Criteria
Inclusion criteria	Age between 55 and 75 years
	Smoking history ≥ 30 pack-years
	Current smokers or former smokers (RISP: cessation ≤ 15 years; ITALUNG2: cessation ≤ 10 years)
Exclusion criteria	Prior diagnosis of lung cancer
	Chest CT performed within the previous 12 months
	Ongoing evaluation for suspicious pulmonary findings
	History of malignancy (except non-melanoma skin cancer)
	Inability to provide informed consent

**Table 2:** Standardized acquisition parameters among all participants.

Parameters	Value
Tube voltage	120 kVp
Tube current	40 mAs
Slice thickness	1 mm
Collimation	≤1 mm
CTDIvol	<2 mGy

**Table 3:** Prevalence of ILAs in our cohort of patients.

LCS Cohort	Patients	ILAs	Male with ILAs	Female with ILAs
RISP	400	28	20	8
ITALUNG2	100	6	3	3
Total	500	34 (6.8%)	23 (67%)	11 (33%)

## Data Availability

The raw data supporting the conclusions of this article will be made available by the authors on request.

## Supplementary Materials

The following supporting information can be downloaded at https://www.mdpi.com/article/10.3390/jcm15093193/s1: Table S1: Visual extension of ILAs according to expert radiologist, less-experienced radiologist and automated software with inter-reader agreement evaluation.

## Abbreviations

The following abbreviations are used in this manuscript:

ILA	Interstitial Lung Abnormality
LCS	Lung Cancer Screening
LC	Lung Cancer
COPD	Chronic Obstructive Pulmonary Disease
CVD	Cardiovascular Disease
LDCT	Low-Dose Computed Tomography
CAC	Coronary Artery Calcifications
