# Supplementary material for: Prevalence of Interstitial Lung Abnormalities (ILAs) in Italian Lung Cancer Screening Programs: A Monocentric Study
Source: J Clin Med. 2026 Apr 22;15(9):3193. doi: 10.3390/jcm15093193 (PMC13164062; doi:10.3390/jcm15093193)
Supplement: Supplementary file 1 [file jcm-15-03193-s001.zip › Supplementary Table S1.pdf]

Table S1. Visual extension of ILAs according to expert radiologist, less-experienced radiologist and automated software with inter-reader agreement evaluation.

N. Patient	Junior radiologist (lung involvement %)	Senior radiologist (lung involvement %)	Automated software (lung involvement %)
**1**	10	10	1
**2**	< 5	< 5	0
**3**	5	5	0
**4**	< 5	< 5	0
**5**	< 5	< 5	0
**6**	5	3	0
**7**	5	5	0
**8**	5	5	0
**9**	5	5	0
**10**	10	5	1
**11**	5	5	0
**12**	< 5	< 5	0
**13**	10	10	1
**14**	10	10	1
**15**	10	5	2
**16**	10	10	1
**17**	5	5	0
**18**	< 5	< 5	0
**19**	5	< 5	0
**20**	5	5	1
**21**	< 5	< 5	1
**22**	5	3	1
**23**	5	5	1
**24**	< 5	< 5	0
**25**	20	20	14
**26**	5	5	3
**27**	< 5	< 5	0
**28**	< 5	< 5	0
**29**	5	5	0
**30**	5	5	1

**31**	< 5	< 5	0
**32**	10	10	2
**33**	5	5	0
**34**	10	10	2
**Inter-observer agreement**		**0.834**	
